# Cancer stem cells in solid tumors: elusive or illusive?

**DOI:** 10.1186/1478-811X-8-6

**Published:** 2010-05-11

**Authors:** Yvonne Welte, James Adjaye, Hans R Lehrach, Christian RA Regenbrecht

**Affiliations:** 1 Department of Vertebrate Genomics, Max Planck Institute for Molecular Genetics, Ihnestrasse 63-73, 14195 Berlin, Germany; 2 Laboratory for Molecular Tumorpathology and Comprehensive Cancer Center Charité, Charitéplatz 1, 10117 Berlin, Germany

## Abstract

During the past years *in vivo *transplantation experiments and *in vitro *colony-forming assays indicated that tumors arise only from rare cells. These cells were shown to bear self-renewal capacities and the ability to recapitulate all cell types within an individual tumor. Due to their phenotypic resemblance to normal stem cells, the term "cancer stem cells" is used. However, some pieces of the puzzle are missing: (a) a stringent definition of cancer stem cells in solid tumors (b) specific markers that only target cells that meet the criteria for a cancer stem cell in a certain type of tumor. These missing parts started an ongoing debate about which is the best method to identify and characterize cancer stem cells, or even if their mere existence is just an artifact caused by the experimental procedures. Recent findings query the cancer stem cell hypothesis for solid tumors itself since it was shown in xenograft transplantation experiments that under appropriate conditions tumor-initiating cells are not rare.

In this review we critically discuss the challenges and prospects of the currently used major methods to identify cancer stem cells. Further on, we reflect the present discussion about the existence of cancer stem cells in solid tumors as well as the amount and characteristics of tumor-initiating cells and finally provide new perspectives like the correlation of cancer stem cells and induced pluripotent cells.

## Review

### Introduction

Already 150 years ago, the German pathologist Rudolf Virchow postulated in his theory of the cellular pathology that cancer initiates from immature cells [1]. But it still took 100 years until Sajiro Makino introduced the term "tumor stem cell" for a small subpopulation of cells that were insensitive to chemotherapy and had chromosomal features different from the bulk of cells [2]. In the 1970s *in vivo *transplantation experiments and *in vitro *colony-forming assays supported Makino's observation that tumors could arise from rare cells with self-renewal capacities. Experiments indicated that these cells are able to recapitulate all cell types within an individual tumor and establish immortal cell lines [3-5].

**Figure 1:**
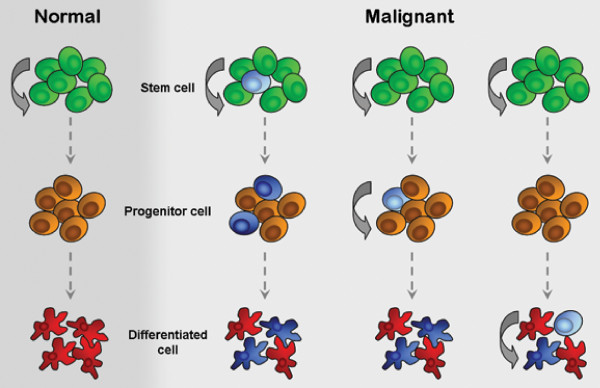
**Origin of Cancer stem cells**. In normal tissue, stem cells (green) divide asymmetrically into progenitor cells (orange) from which then terminally differentiated cells (red) are produced (left). In tumorigenesis mutations can transform stem cells into cancer stem cells (light blue) which then result in tumorigenic progenitor cells and differentiated tumor cells (dark blue). But also, by mutations in developmental pathways progenitor cells and differentiated cells can re-acquire stem cell-like properties and turn into cancer stem cells (right).

These so called cancer stem cells (CSC) have been proposed to originate either from malignant transformation of a normal somatic stem cell or a progenitor cell [6] (Figure 1). Since stem cells proliferate throughout life they are more susceptible to accumulate oncogenic mutations than differentiated cells with their comparatively short life span [7,8].

On the other hand, it could be that differentiated cells reacquire stem cell-like characteristics by the reactivation of signaling pathways like the Wnt-beta-catenin and Bmi1 pathway or certain Hox genes that facilitate self-renewal and are linked to malignant transformation of cells [9-11].

One of the great advantages of the cancer stem cell hypothesis is that it also helps understanding other cancer concepts such as cancer as a minimal residual disease. Even a single cell that evades the surgeon's blade or adjuvant therapies by acquired resistance like effective DNA repair mechanisms or high amounts of active ABC transporters that rapidly efflux chemotherapeutics recapitulates the whole tumorigenesis resulting in a relapse after what seems like a successful cancer treatment.

If the cancer stem cell hypothesis holds true at least for some tumor entities, this calls for new pharmacological perspectives to efficiently target these cells to prevent relapse and metastasis. While specific cell surface markers for CSCs in hematological malignancies are widely accepted and the concept of only a rare subpopulation of cancer cells that exhibit stem cell-like characteristics and promote growth of hematological tumors is far beyond any doubt, the situation looks totally different in solid tumors. Whereas some scientists still argue about the best method to identify and characterize CSCs, others already query the CSC hypothesis in solid tumors itself since it was shown in xenograft transplantation models that under appropriate conditions tumor-initiating cells are not rare [12]. Much of the confusion in the field comes from the varying definitions of cancer stem cells. For example, asymmetric cell division vs. self-renewal: any cell that divides to give two daughter cells identical to the parental cells has self-renewed, and presumably the majority of cells within a tumor that are dividing self-renew in this sense. Thus, there must be more to a definition of a cancer stem cell than self-renewal. The key is whether a cell can yield multiple different sub-populations cells within a tumor that found a hierarchy within the tumor by asymmetric cell division.

In this review we discuss the prospects and challenges of the currently used main methods to identify CSCs. Further on, we reflect the present discussion about the existence of CSCs as well as the amount and characteristics of tumor-initiating cells and finally provide new perspectives like the correlation of CSCs and induced pluripotent cells.

### Current standards of identification

The identification of a putative cancer stem cell subpopulation with validated methods and markers for each tumor entity remains controversial. So far, researchers take advantage of known stem cell characteristics like the ability to self-renew, expression of stem cell markers and their multipotency. The most widely accepted assays to validate a candidate cancer stem cell subpopulation are efflux analysis of the DNA-binding dye Hoechst as well as detection of known stem cell markers in cancer cells and verification by xenotransplantations.

### Cell surface markers

In the last decade several molecular properties have been utilized to identify and characterize CSCs from different hematopoietic and solid tumors. The first markers used were cell surface proteins already known to define stem and progenitor cells, e.g. CD133 and CD166. Furthermore, molecules that facilitate drug resistance in cancer cells like ABCB1 and ABCG2 were added to the list of putative CSC markers as well as proteins for which no involvement in stemness or cancerogenesis was known, e.g. CD20. In the following section we discuss examples of proposed markers which were mostly used to identify CSCs of solid tumors in the past.

CD133 (also known as Prominin 1), a member of pentaspan transmembrane glycoproteins, is expressed in hematopoietic stem cells, endothelial progenitor cells, neuronal and glial stem cells [13-15] and specifically localizes to cellular protrusions [16]. CD133 has previously also been shown to be expressed in subpopulations of cancer cells from brain, colon, lung, melanoma and other solid tumors. This led to the assumption that CD133 expressing tumor cells have stem cell or progenitor like properties and CD133 was proposed as CSC marker [7,17-19].

To illustrate the correlation of CD133 expression with the level of differentiation of a certain cell type, the group of Huttner generated an antibody that recognizes CD133 independently of potential posttranslational modifications as glycosylation. They found that the expression of CD133 is independent from the cell's state of differentiation, while posttranslational glycosylation negatively correlates with differentiation [20]. Only AC133, the glycosylated epitope of CD133, is downregulated upon cell differentiation. Therefore it seems likely that upon dedifferentiation of cells as observed in oncogenesis the glycosylation of CD133 (AC133) might also increase and serve as a marker for the tumorigenic potential of a cell.

Keeping this in mind, one has to be cautious when interpreting results from experiments where it is unclear if the antibody detected CD133 or AC133 as many groups in the field seem to use the term CD133 synonymously to AC133. This inattentiveness could lead to confusions in interpreting the results.

In 2007 Klein *et al*. have observed an increased expression of CD133 in primary and metastatic melanoma compared to melanocytic nevi [21]. Subsequently, other groups found that also in a variety of tumors such as hepatocellular and rectal cancer an increased CD133 expression corresponds with higher stage tumors and poor prognosis [22,23].

Previously, disseminated tumor cells of melanoma patients with metastatic disease have been shown to express stem cell markers CD133 and NESTIN [24]. Those disseminated tumor cells are currently under debate of being involved in the formation of metastases and correlation with poor prognosis [25]. However, beyond any doubts is the anchorage-independent growth of these disseminated tumor cells which is also a characteristic of stem cells from various types of self-renewing tissue [26,27].

In cell culture experiments, antibody reactivity against CD133 has been shown to correlate with the cell cycle DNA profile of colon cancer, melanoma, and human embryonic stem cells. Cells with highest ectopic expression of CD133 had a DNA content of 4N or even greater and reflect cycling cells [28]. These findings are concordant with the results of Grskovic and Liu that CD133^+ ^cells have a higher mitotic index compared to CD133^- ^cells in the first week of cultivation [29,30].

Moreover, Liu *et al*. showed that CD133 expression is significantly higher in recurrent glioblastoma tissue as compared to their respective primary tumors. Additionally, CD133^+ ^cells in three primary cultured cell lines established from glioblastoma patients showed an increased expression of proteins associated with neural precursors, e.g. CD90, NESTIN and MSI1 compared to autologous CD133^- ^cells as well as higher levels of ABCG2 and the DNA repair protein MGMT and higher mRNA levels of anti-apoptotic genes. For the first time ever, this study provided evidence that these properties contribute to the tumor's resistance to chemotherapy. CD133^+ ^CSCs were significantly more resistant to chemotherapeutic agents compared to autologous CD133^- ^cells [30].

CD133^+ ^cells within human osteosarcoma cell lines as well as human melanomas were also shown to have many CSC like properties as for example formation of sphere-like colonies after cultivation under serum-free conditions [7,31].

Finally, CD133^+ ^cells of various tumor entities were shown to have an increased tumorigenic potential. For melanoma this was demonstrated for the first time by Monzani and collegues. Magnetically sorted CD133^+ ^and CD133^- ^cells respectively, were injected into NOD-SCID mice. After 40-50 days, mice injected with CD133^+ ^cells developed detectable tumors, whereas mice injected with CD133^- ^melanoma cells did not develop neoplasia even 4 months after injection [7].

In addition to its role as a cancer stem cell marker, CD133 could also serve as an important therapeutic target for metastatic melanoma and potentially for other CD133 expressing cancer types. In 2008, Rappa *et al*. investigated the effects of CD133 down-regulation in human metastatic melanoma, which result in slower cell growth, reduced cell motility, decreased capacity to form spheres under stem cell-like growth conditions and reduced capacity of the cells to metastasize. Monoclonal antibodies directed against two different epitopes of the CD133 protein induced a specific, dose-dependent cytotoxic effect. In cells with only residual CD133 expression, microarray analysis revealed expression changes for 143 annotated genes. 13% of the up-regulated genes coded for Wnt inhibitors [32]. In it's normal function Wnt signaling is crucial for tissue homeostasis and self-renewal in a variety of adult tissues as well as embryonic development and hematopoesis [33,34]. Additionally Wnt has been implicated as being one of the drivers of oncogenesis in various organs [35-37].

However, previous experiments revealed that some tumor cells, which do not express CD133, are also capable of self-renewal and are tumorigenic. For human gliomas Wang *et al*. demonstrated that CD133^- ^cells derived from 6 different patients were tumorigenic when implanted into brains of nude rats. For 3 of these patients, analysis showed that the resulting tumors contained CD133 positive cells [38].

Even more contradictory are the results in C6 glioma cells: Whereas Zhou *et al*. demonstrated that this cell line contains only a small fraction of cells that can form tumor spheres in serum-free stem cell medium and express stem cell markers CD133 and NESTIN [39], Zheng *et al*. concluded that the C6 line is mainly composed of CSCs, although many of them are CD133^- ^[40]. Each of the tested single C6 cells was able to generate a clone and subclones in serum-containing medium, which subsequently gave rise to a xenograft glioma in nude mice. The latter group confirmed these results the following year showing that most C6 cells are cancer stem-like cells with characteristics of self-renewal, multilineage differentiation potentials *in vitro*, and tumorigenic capacity *in vivo *irrespective of their CD133 expression [41].

CD133^+ ^and CD133^- ^cells of lung cancer were also examined for their abilities of colony formation, self-renewal, proliferation, differentiation and invasion, as well as resistance to chemotherapeutic drugs. The results suggested that both the CD133^+ ^and CD133^- ^subpopulations contain similar numbers of cancer stem cells since they displayed similar abilities [42]. Analogical results were obtained for human metastatic colon cancer cells [43].

Despite the fact that not all groups concurrently used the AC133 antibody to isolate CD133^+ ^cells, the choice of antibody would explain these contradictory findings only to some extent. Even research groups that investigated the same cell type with the same type of antibodies arrived at different conclusions regarding the use of CD133/AC133 as CSC marker [39,41]. Taken together, CD133/AC133 is an indicator, but definitely not a reliable marker for defining a population of CSCs in solid tumors since it does not characterize tumor-initiating cells exclusively. Therefore CD133/AC133 should be seen as a necessary however insufficient criterion to identify CSCs in solid tumors.

Comparably controversial results as for CD133 were obtained from the investigations of various ATP-Binding Cassette (ABC) transporters. Schatton *et al*. described tumor-initiating cells capable of self-renewal and differentiation in human melanoma defined by expression of the chemoresistance mediator ABCB5. ABCB5 expression in tumor cells correlates with clinical melanoma progression. In serial xenotransplantation experiments ABCB5^+ ^melanoma cells were more tumorigenic than ABCB5^- ^cells. Additionally, ABCB5^- ^cells showed no differentiation capacity since they exclusively gave rise to ABCB5^- ^cells whereas ABCB5^+ ^cells regenerated both subpopulations. Using a monoclonal anti-ABCB5 antibody in nude mice, initial tumor growth as well as growth of established tumors was inhibited by antibody-dependent cell-mediated cytotoxicity in ABCB5^+ ^cells [44]. Monzani *et al*. identified a subpopulation of human melanoma cells co-expressing ABCB1, ABCB5 and ABCC2 in addition to stem cell markers which demonstrated higher clonogenicity, self-renewal capacity and anchorage-independent growth than the negative fraction [45]. Furthermore, they identified tumor-initiating cells in human melanoma by the expression of ABCG2 which is coexpressed with CD133 [7]. For colon, breast and prostate cancer cell lines however, these results could not be confirmed [46]. Patrawala *et al*. compared ABCG2^+ ^and ABCG2^- ^cancer cells with respect to their tumorigenicity *in vivo*, but no significant difference in tumor incidence or latency periods comparing the two populations was observed [46]. Finally, Quintana *et al*. examined the expression of more than 50 surface markers on melanoma cells and injected these cells into NOD/SCID mice lacking the interleukin-2 gamma receptor. In every case, tumors arose from all fractions of cells. No known marker distinguished tumorigenic from non-tumorigenic cells [12].

Concluding these results, it seems as if tumor-initiating cells are phenotypically heterogeneous and no marker or set of markers has been found to identify CSCs in solid tumors in general nor for specific tumor entities.

### Dye exclusion assays

In contrast to preferably cell-type specific surface markers, the use of Hoechst-dye to identify and isolate CSCs as a so called side population (SP) overcomes the barrier of phenotypical markers and replaces it by more direct functional markers [47]. The blue fluorescent Hoechst 33342 is a cell permeable bisbenzimidazole derivative that binds to the minor groove of DNA. After excitation of Hoechst its emission can be measured simultaneously in the blue and red spectrum. But although Hoechst enters viable cells, it is also actively pumped out by ATP-Binding Cassette (ABC) transporters of the cell membrane [48-50]. Goodell *et al*. were the first to identify that hematopoietic stem cells are particularly effective at pumping out Hoechst [51] since they express high levels of ABC transporters resulting in a small side population of weakly stained cells which can be observed during flow cytometric analysis. To determine the size of the side population, verapamil, an L-type calcium channel blocking agent serves as an important control. Blocking the calcium channels inhibits the efflux of Hoechst-dye from these cells, so it is then possible to gate for the side-population, which is suspected to consist of cancer stem cells. Subsequently, side populations were identified in various established cell lines from breast cancer, lung cancer and glioblastoma, suggesting that this phenotype defines a class of cancer stem cells with inherently high resistance to chemotherapeutic agents due to rapid efflux of those compounds [52]. Kondo *et al*. were the first to come up with the hypothesis that the side population resembles the source of CSCs in C6 glioma cells since only SP cells initiated tumors in multiple tissues in nude mice [53]. Also, purified side population cells from breast cancer and thyroid cell lines showed higher tumorigenicity than corresponding non-side population cells. They have a differential gene expression profile and preferentially express genes related to stemness, including *NOTCH1 *and *CTNNB1 *at higher levels [46,54]. Grichnik *et al*. identified side population cells in metastatic melanoma cell lines which, compared to non-side population cells were small in size, less melanized, had a decreased proliferation rate and gave rise to a heterogeneous cell population [55]. All these findings support the isolation of side populations via Hoechst staining as an identification method for CSCs. Additionally, this method could help to identify more specific molecular CSC markers by comparing the expression profiles of SP and non-SP cells which is crucial for the establishment of targeted cancer therapies.

The main criterion of CSCs in contrast to non-CSCs is their unique capability to differentiate and recapitulate all cell types within a tumor. The results of several groups led to the conclusion that the analysis of a SP via Hoechst staining is a useful technique to isolate putative CSCs. They demonstrated that only SP cells generate both SP and non-SP cells in cell culture while non-SP cells fail to do so [52,53,56,57]. Furthermore, they were able to initiate tumors in xenograft transplantation experiments with very low numbers of SP cells, whereas non-SP cells were either not at all tumorigenic or only upon injection of multifold more cells compared to the SP.

In contrast to these encouraging findings, results from studies with thyroid, gastrointestinal, adrenocortical and glioma cancer cells question the possibility to identify CSCs by their efflux-capacity. They depict very well that non-SP cells are able to generate SP cells, have similar growth rates and tumor-initiating capacity as SP cells [54,58-60].

The same controversial results were obtained regarding the expression of specific stem cell/CSC markers on SP cells. Although it was previously shown that intestinal epithelial stem cells can be isolated as a side population (SP) by FACS after staining with Hoechst [61] this concept did not apply to gastrointestinal cancer cells tested by Burkert *et al*. [58]. SP cells of several gastrointestinal cancer cell lines showed no increased expression of stem cell markers like CD133, CD44, Musashi-1, Oct-4 and CD117 compared to non-SP cells. Both fractions were similarly clonogenic *in vitro*, tumorigenic *in vivo*, and displayed similar differentiation potential *in vitro *and *in vivo*.

ABC transporters that most notably account for the efflux of Hoechst are ABCG2 (Bcrp1) and Mdr-1 (also known as P-glycoprotein or ABCB1) [48,50,51,62,63]. This is concordant with the findings that these genes are highly expressed on SP cells but not on non-SP cells [46,52]. Congruously, putative CSCs in the SP have greater capacity to expel cytotoxic drugs used in cancer therapy, therefore improve their survival and finally recapitulate the whole tumorigenesis resulting in a relapse after what seems like a successful cancer treatment [52,64].

However, expression levels of ABCG2 and Mdr-1 was shown to be identical on non-SP and SP cells in gastrointestinal cancer indicating that there may be additional factors responsible for the Hoechst effluxing property [58]. Furthermore, other studies showed that side population cells obviously express some transporter molecules responsible for Hoechst efflux. But this alone seems to be insufficient to ensure chemotherapy resistance associated with a survival benefit over non-SP cells [59].

A good example of the influence of technical factors on the results is the comparison of studies from Kondo *et al*. and Patrawala *et al*. Both groups studied the same cell lines, but the yield of SP cells varied among the magnitude of 10 [46,53].

Due to these controversial findings some scientists argue that Hoechst staining and isolation of SP cells cannot be applied to identify and isolate CSCs, at least for some tumor entities.

We think these controversial results are mostly due to inefficient and lenient sorting procedures that never result in 100% pure CSC and non-CSC fractions. Variations in the staining protocol and FACS procedure can have enormous influence on the yield, viability and homogeneity of side population cells, affecting all later analyses done with these cells. In order to gain reproducible results, tissue dissociation to single cell suspension levels and cell counting need to be optimized. Optimal Hoechst concentration should be independently determined for each new tissue studied and the optimal Hoechst concentration should be within the plateau region [65]. But Hoechst concentration is exactly the parameter that mostly differs among published data. Used concentrations ranged from 2-10 μg/ml and the incubation time from 30-120 minutes [53,55,66-71].

Furthermore, it has been shown that various cell types are unequally sensitive to verapamil which serves as an important control. Nethertheless, most groups apply a concentration of 50 μM verapamil without determining the cell-line specific sensitivity prior to the SP sorting [46,55,67].

The reasons to explain the differences between Kondo *et al*. and Patrawala *et al*. range from the use of different flow cytometers and modified protocols [46]. Indeed, Patrawala's group used higher Hoechst concentrations than Kondo and according to studies from Kunkel higher Hoechst concentrations result in a smaller SP [53]. Patrawala *et al*. therefore legitimately reasoned that their experimental setup was more stringent for the identification of putative CSCs [46].

In addition to the technical aspects of FACS sorting, it was demonstrated that the SP size depends on the density of cells in culture. SP cells preferentially survive at very low plating density, because such clonal growth favors the presence of CSCs [54].

Due to the range of parameters that can dramatically influence SP analyses, results from such sorting experiments should be compared very critically. Optimized and standardized protocols for each cell type as well as stringent cell culture and isolation settings are required to eliminate the risk of analyzing different SP cells. These standards will help to abolish skepticism and uncertainty about the general validity of the technique and potential of SP cells.

Ultimately, the toxicity of Hoechst should be addressed and always kept in mind when applying this dye to isolate putative CSCs. As Hoechst binds to DNA, it can disrupt DNA replication during cell division. Consequently, it is potentially mutagenic and carcinogenic. What causes the researcher to adhere to strict safety regulations while working with Hoechst, also affects all cells that are stained *in vitro*. Already in 1986, Siemann *et al*. evaluated the toxicity of this stain in cells derived from sarcomas. Hoechst toxicity increased with increasing exposure times resulting in 25- to 45-fold reduced survival in irradiated cells and 4- to 5-fold reduced survival in untreated cells. Furthermore, cytotoxic effects of Hoechst 33342 were found to be significantly greater in cells in the S phase than in cells in G1 and G2-M phases of the cell cycle [72]. Recently, these results were confirmed by Shen *et al*. They showed that Hoechst staining leads to obvious morphological alterations and increased apoptosis in the C6 glioma cell line [41]. This toxicity, particularly its cell cycle specificity, suggests a potentially severe limitation for the use of Hoechst dye in combination with fluorescence activated cell sorting. Non-SP cells with lower capacity to efflux this dye will suffer more from its effects compared to putative CSCs of the SP. Hence, Wu *et al*. argued that SP cells may not represent stem-like cells, but rather, a population of cells that is able to escape the lethal effects of Hoechst staining [73].

An alternative to the use of Hoechst provides the use of Rhodamine 123 which was shown to be non-toxic to cells even at high concentrations [74]. The cell-permeable, green-fluorescent Rhodamine 123 binds to mitochondrion membranes. Like Hoechst it is actively pumped out of the cells by ABC transporters, e.g. MDR1 and ABCB1 [75,76]. Liu *et al*. compared the use of Rhodamine 123 and Hoechst to isolate CSCs in a hepatocellular cell line. The percentages of SP and non-SP after staining with Rhodamine 123 or Hoechst were the same as well as the proliferative abilities *in vitro*, expression of stem cell markers and tumorigenicities *in vivo *of both obtained side populations [77]. Taken together, use of Rhodamine 123 in combination with flow cytometric cell sorting may be a useful method for CSC identification.

### In vivo transplantation experiments - are CSCs rare cells?

Until today the gold standard in validating a putative CSC fraction is their transplantation into immunodeficient mice. If CSCs are really enriched in this population, these cells should have a several fold higher capacity to form tumors compared to the control fraction where the cells lack this CSC marker or the typical characteristics as rapid efflux of Hoechst due to high expression of ABC transporters.

In this manner, the side population after Hoechst staining and flow cytometric analysis was proved to be enriched of tumor-initiating cells in thyroid [54], ovarian [78] and breast cancer [46], glioma [46,53], melanoma [70] and hepatocellular carcinoma [79]. Further on, xenotransplantation experiments validated certain CSC markers like ABCB5 for melanoma [44], CD133 for melanoma [7], lung [19] and colon cancer [18,80] and CD20 for melanoma [81].

Until two years ago, it seemed that only few cells isolated from a tumor have the ability to initiate tumor growth in transplantation experiments. But in 2008 Quintana *et al*. put this dogma of rare CSCs at least for melanoma into question. They found out that melanoma-initiating cells are only rare in NOD/SCID mice if monitoring tumor formation for a short term, like most researcher do, and using the common assays described in literature.

But this frequency could be significantly increased by following melanoma formation for more than 8 weeks, using NOD/SCID IL2Rγ^null ^mice that lack natural killer cell activity compared to NOD/SCID mice and injecting melanoma cells together with Matrigel, a mixture of structural proteins and growth factors. Overall, after injection of single, unselected melanoma cells, 27% initiated a tumor suggesting that so far the frequency of melanoma-initiating cells was significantly underestimated [12].

With this modified assay Quintana *et al*. provided a good example of the importance of interactions between tumor cells and their extracellular matrix and how they dictate whether or not a tumor develops from a mutated cell.

Matrigel represents a rich store of matrix proteins as well as angiogenic and growth factors which promote tumor growth and metastasis [82]. But still the aberrant extracellular matrix of immunodeficient mice cannot substitute for all factors of the human microenvironment, and the stem cell niche which is crucial to initiate tumor growth is not reconstituted in xenotransplantation models [83].

This is supported by studies that show that the frequency of cells that sustain tumor growth was dramatically increased using allograft transplantation models. Kelly *et al*. transferred mouse lymphoma cells into non-irradiated histocompatible recipient mice. Regardless of cell number injected, all animals developed a tumor even when transplanting only a single neoplastic cell [84]. Additionally, studies using spontaneously arising tumors in syngeneic rodents have shown that the number of cells required to transplant a tumor depends on the site of transplantation and the type of cells [85].

Last but not least, the amount of tumor-initiating cells is influenced by the experimental setup. Enzyme treatment during the preparation of the cell suspensions and sorting procedure might decrease the viability of tumor cells and modify protein expression, thereby affecting the population into which the cells are sorted and the ability of these proteins to play a role in tumor formation following transplantation [86].

But instead of focusing on the establishment of methods to increase the percentage of tumor-initiating cells in artificial xenograft transplantation models with immunosuppressed mice, should we not answer the question which cells initiate tumors in healthy organisms with intact immune systems as well as resist cancer therapies and causing relapses, respectively? In 2009 Schatton and Frank proposed that CSCs in human melanoma that express the chemoresistance mediator ABCB5 might be responsible for melanoma immune evasion [87]. Thus, immunomodulation might represent the key mechanism by which CSCs promote tumorigenic growth.

### Cancer stem cells, quo vadis?

Models serve the purpose of simplifying complex contexts. Traditionally, signaling cascades are depicted as straight forward processes in a highly ordered manner. This way of looking at biological systems started a true gold rush in the field of stem cell marker identification. So, in the past much effort was made to identify novel key cancer stem cell markers for specific tumor entities, if not a universal marker or set of markers for CSCs in all cancers. But in reality the biology with all its feedback loops, branching, positive and negative modulators is much more complex. This makes it a challenge to identify the right components that uniquely define cancer stem cells.

On the other hand standards are needed to help interpreting the results of various groups working on the elucidation of CSC nature. But into which direction should it be leading to? Which methods, which protocols are going to be the right ones?

One approach for the characterization of CSCs that was not considered yet, could be the analysis of analogies between cancer stem cells and induced pluripotent stem (iPS) cells.

In 2006 Takahashi and Yamanaka were the first who successfully reversed differentiated mouse embryonic or adult fibroblasts into pluripotent stem cells by introducing only the four factors Oct3/4, Sox2, c-Myc and Klf4 and named these reprogrammed cells which exhibit the morphology and growth properties of embryonic stem (ES) cells and express ES cell marker genes, induced pluripotent stem cells [88].

After inducing somatic cells with these four "Yamanaka factors", Mikkelsen and colleagues found out that about 20% of the cells stained positive for the stem-cell marker SSEA1, but only about 1.2% were fully reprogrammed. Whereas fully reprogrammed cells showed gene expression and epigenetic states similar to that of ES cells, gene expression profiling revealed that partially reprogrammed cells re-activated only a small group of genes related to stem cell renewal and maintenance, but yet these cells are not pluripotent. Instead they exhibit a down-regulation of structural genes and regulatory factors expressed in differentiated cells and up-regulation of some lineage-specific and proliferative genes not expressed in iPS cells [89].

**Figure 2:**
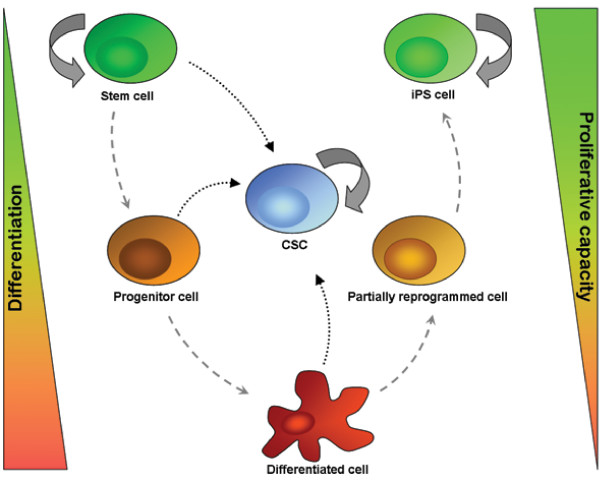
**Relationship between stem cell species**. Stem cells are characterized by their ability to form many different types of tissues and their capacity to self-renew. With increasing level of differentiation from progenitor cell to differentiated cell, the plasticity reduces as does the proliferative capacity. Cancer stem cells form at the interface between stem cell and progenitor cells. This phenomenon has lately also been credited to iPS cells and their partially reprogrammed precursors.

Potentially, the same is true for cancer stem cells. Probably they represent an intermediate state between stem cells and differentiated cells like partially reprogrammed cells do between iPS cells and differentiated cells (Figure 2). Mikkelsen *et al*. showed that these differences are due to epigenetic events like persistent DNA hypermethylation at pluripotency- and germ-cell-specific loci [89].

In addition, it is known that small molecules like the histone deacetylase inhibitor valproic acid, the DNA methyltransferase inhibitor 5-aza-cytidine or the kinase inhibitor Kenpaullone facilitate iPS cell formation by modeling epigenetic information [90-92].

The comparison of melanoma cells enriched for cancer stem cells and non-cancer stem cells in our lab also argues in favor of epigenetic regulation and maintenance of the phenotype or alternative splicing since we found no differences in coding mutations at the genome or transcriptome level but tremendous varieties among gene expression patterns (unpublished data).

This is also consistent with the plasticity of cancer because only epigenetic modulations can efficiently be activated and reverted in short intervals. This model has been recently discussed by Gupta *et al*. They postulated that there might exist a dynamic equilibrium between CSCs and non-CSCs within tumors that may be shifted in one direction or another by contextual signals within the tumor microenvironment that influence the probability of interconversion between the CSC and non-CSC compartments [93]. This would also explain the varying amounts of cancer stem cells within the same tumor entity described by different groups.

Evidence for a conversion of CSC into non-CSCs and vice versa were given by Platet *et al*. and Lichtenauer *et al*. Both groups found that non-SP cells in flow cytometric analyses after Hoechst staining are able to generate SP cells [59,60]. That means bulk tumor cells can convert into CSCs and explains the maintenance of the SP phenotype in long-term cell cultures. This dynamic equilibrium was also seen within the partially reprogrammed cells in the group of Jaenisch where cells positive for SSEA1 as well as cells negative for this stem cell marker reverted to the heterogeneous state within 1-2 passages in cell culture [89].

But still one question remains unsolved. How is this equilibrium driven and which cells transmit the required signals? Are the non-CSC alone really able to de-differentiate into CSC or do they obtain the priming signals to reconstitute the balance within the tumor microenvironment from the small part of CSCs. MicroRNAs probably play a role in facilitating the equilibrium of CSCs and non-CSCs like they promote iPS cell formation by their regulatory effects on epigenetic and transcriptional modulation [94]. We should learn from this quite new but extensively investigated research field and try consigning these new insights to the CSC research.

One - of course provocative - interpretation of the seemingly contradictive findings on CSCs is that cancer stem cells are neither a defined nor a definable sub-population of cells within the tumor, but rather a highly dynamic state in which only few cells at a time are in. We learn from Heisenberg's "uncertainty principle" that in a single experiment only the position (= state) or speed (= change over time) can be determined with any given precision. This principle does not only hold true for quantum mechanics, but also applies to observation made on living cells. For example by sorting the cells for their stem cell-like properties, the result is only a snapshot of the cells' state, but we cannot deduce from this observation that also the bulk cells cannot acquire such properties. On the other hand, if we follow the dynamic changes in the level of markers, we cannot determine whether this comes from an increase in cells expressing the marker, or if the expression level per cell has increased. Keeping this in mind one has to be very careful when interpreting the current literature on cancer stem cells.

## Conclusions

Since Nixon's war on cancer billions of dollars were invested in cancer research. But still, our understanding of the biology of cancer does not help to cure this dreadful disease. The concept of cancer stem cells was very welcome because it opened new perspectives in understanding and ultimately healing this disease.

Although it is tempting to explain tumor formation and metastasis by the presence of stem cells, after almost a decade of intense research, it seems that cancer stem cells still do not explain how neoplasias evolve. The inconsistencies in the experiments call for a more in depth analysis of the cellular and signaling features of those cells. The research therefore must focus on two things: (a) the establishment of robust and reliable criteria to identify and isolate cancer stem cells and (b) in parallel researchers must find and agree on an unbiased definition of what a cancer stem cell really is. This may be the activation/deactivation of specific pathways or the presence/absence of expression of proteins that discriminate them from other cells within the tumor.

In the worst case, many of the observations made in cancer stem cells would be nothing but artefacts, which are induced by the researcher by the artificial environment that is presented to the cells in form of the conditions under which the cells are cultured. But even then, the existence of subpopulations of cells with unique features helped to make researchers more sensitive towards the heterogeneity not only within a tumor *in vivo*, but also in cell culture models *in vitro*.

From what we know about cancer stem cells today, we would not expect the worst case to become true. It seems more and more likely that this population of cells is not a defined group of cells resting in a niche and populating the tumor with amplifying cells, but that few or maybe even many cells within the tumor can function as cancer stem cells if induced, but also can go back to the state of a "normal" cancer cell. Of course this scenario makes it even more difficult to target these cells for therapeutic reasons. Although, research has shed some light on the matter, the question still remains unanswered: Are CSCs in solid tumors elusive, or illusive?

## Competing interests

The authors declare that they have no competing interests.

## Authors' contributions

YW and CRAR contributed to the writing and conceptual design and preparation the figures of this review. All authors have read and approved the final manuscript.
